# Mercury exposure in ringed seals (*Pusa hispida saimensis*) in Lake Saimaa, Finland, and the placenta as a possible non-invasive biomonitoring tool

**DOI:** 10.1007/s11356-024-34980-6

**Published:** 2024-09-18

**Authors:** Jesse Simola, Mervi Kunnasranta, Marja Niemi, Vincent Biard, Jarkko Akkanen

**Affiliations:** 1 Department of Environmental and Biological Sciences, University of Eastern Finland, P.O. Box 111, FIN-80101 Joensuu, Finland; 2 Natural Resource Institute Finland, Yliopistokatu 6, FIN-80100 Joensuu, Finland

**Keywords:** Ecotoxicity, Endangered, Heavy metal, Neurotoxicity, Pinniped, Prenatal exposure, Contamination, Selenium

## Abstract

The Saimaa ringed seal (*Pusa hispida saimensis*) is a subspecies of ringed seal, landlocked in Lake Saimaa, Finland. The small population of less than 500 seals is facing many human-induced threats, including chemical contaminants. Mercury, in particular, has previously been suggested to be one of the chemicals affecting the viability of this endangered population. We analysed mercury concentrations from placentas and lanugo pup tissues (blubber, brain, kidney, liver, and muscle) to determine current prenatal exposure levels. These pups were found dead in or near birth lairs and were less than 3 months old. Additionally, we used threshold values available in the literature to estimate the potential mercury toxicity to the Saimaa ringed seal. We also determined selenium concentrations for its potential to alleviate the adverse effects of mercury. We further supplemented our study with brain samples collected from various seal age classes. These seals were found dead by either natural causes or by being caught in gillnets. The analysed chemicals were present in all tissues. For lanugo pups, mercury concentrations were the highest in the kidney and liver, whereas the highest selenium to mercury molar ratio was observed in placentas. The toxicity evaluation suggested that, in severe cases, mercury may cause adverse effects in lanugo and older pups. In these cases, the selenium concentrations were low and selenium to mercury ratio was below 1:1 threshold ratio and thus unlikely to provide adequate protection from the adverse effects of mercury. Furthermore, adverse effects are more likely to occur in adult seals, as mercury bioaccumulates, leading to higher concentrations in older individuals. Placental mercury concentrations correlated to those in the livers and muscle tissues of lanugo pups. This, together with the fact that placentas can be collected non-invasively and in good condition, provides a potential novel method for biomonitoring mercury exposure in Saimaa ringed seals.

**Supplementary Information:**

The online version contains supplementary material available at 10.1007/s11356-024-34980-6.

## Introduction

Mercury is a potent neurotoxin that bioaccumulates and biomagnifies in aquatic food chains, especially in its organic form of methylmercury (MeHg) (Fant et al. 2001; Brookens et al. 2008; Luoma and Rainbow 2008; Bridges and Zalups 2010). Apex predators have a particularly high risk for mercury bioaccumulation (Luoma and Rainbow 2008), and numerous studies have revealed elevated concentrations in freshwater (Siimes et al. 2019) and marine fishes (AMAP 2002), cetaceans (Rawson et al. 1993; AMAP 2002), and pinnipeds (Kari and Kauranen 1978; Brookens et al. 2008). Due to their high position in food webs, pinnipeds have been the concern of mercury studies for decades (e.g. AMAP 2011, 2018; Dietz et al. 2021), and mercury has been observed in both marine (AMAP 2002, 2018) and freshwater seals (Kari and Kauranen 1978; Trukhanova et al. 2022).

The endangered Saimaa ringed seal (*Pusa hispida saimensis*) is a subspecies of ringed seal landlocked in Lake Saimaa, Finland. The population faces multiple threats that include a small population size with a narrow gene pool, habitat degradation through climate change, by-catch mortality, and anthropogenic contamination (Kunnasranta et al. 2021; Sundell et al. 2023). The area around Lake Saimaa is industrialised, with notable forest-derived industries. This has increased the mercury exposure to the lake ecosystem, as mercury was used as an antifoulant agent during pulp processing until the 1970s, after which, its use was banned. Despite legislative efforts (Regulation 2017/852 of the European Parliament and of the Council) to decrease mercury pollution, mercury contamination is still detectable today in common fish species in Finland (Siimes et al. 2019). For instance, the mean mercury concentrations in perches (*Perca fluviatilis*) from different regions of Lake Saimaa varied between 0.03 and 0.43 mg/kg wet weight (ww.). The highest concentrations are close to the upper limit for safe consumption (0.5 mg/kg ww.) stated by the European Commission regulation (1881/2006). Nowadays, long-range atmospheric transport and environmental change (land-use change, climate change) contribute to the mercury burden.

Mercury has been suggested as one reason for the population decline of the Saimaa ringed seal during the1960s and 1970s as well as for the stillbirths due to prenatal exposure in the 1980s (Hyvärinen and Sipilä 1984; Hyvärinen et al. 1998). Between 1982 and 1984, over 19% (Sipilä et al. 1990) of pups died soon after delivery, and some 30 years later, the average perinatal mortality remained over 13% during 2011 to 2013 (Auttila et al. 2014). Since mercury has been proposed as a factor causing perinatal mortality (Hyvärinen and Sipilä 1984), there has been continued interest in assessing the mercury levels in Saimaa ringed seals. Although Kari and Kauranen (1978) reported a staggering total mercury (THg) concentration reaching up to 510 mg/kg ww., the mean mercury concentration in adult Saimaa ringed seal livers has decreased since the 1960s (Helminen et al. 1968; Kari and Kauranen 1978; Hyvärinen et al. 1998). This is further confirmed by Lyytikäinen et al. (2015), who observed a net decline in the THg liver concentrations of adult seals, with a mean of 69 mg/kg ww. Nonetheless, the prevalences of stillbirth and pre-weaned pup mortalities have remained relatively high (Auttila et al. 2014), which may, in part, be attributed to mercury exposure (Lyytikäinen et al. 2015).

As mercury has a high potential for causing adverse effects in the environment, studies commonly include selenium concentrations due to its capability to alleviate the possible harmful effects of mercury (Luoma and Rainbow 2008; Zwolak and Zaporowska 2012; Das et al. 2016; Gerson et al. 2020). This protective effect occurs when selenium binds with mercury and forms, for example, biomineral tiemannite (Nigro and Leonzio 1996; Khan and Wang 2009), which has been observed in the livers of Saimaa ringed seals (Lyytikäinen et al. 2015). Generally, a selenium to mercury (Se:Hg) molecular ratio of 1:1 or higher in favour of selenium is considered sufficient to mitigate mercury’s toxic effects (Ralston et al. 2007; Sørmo et al. 2011; Mulder et al. 2012), but Gerson et al. (2020) have questioned this generalisation. As they noted, selenium cannot provide protection in all instances. For example, in an in vitro study by Das et al. (2016), selenium was unable to protect harbour seal (*Phoca vitulina*) immune cells from methylmercury toxicity. In another study by Cabazas-Sanchez et al. (2019), selenium actually increased the bioaccumulation of mercury in the brain and liver of zebrafish (*Danio rerio*). In addition to being unable to provide protection from the effects of mercury, selenium itself may be a source of toxicity (Zwolak and Zaporowska 2012; Gerson et al. 2021). However, selenium concentrations are generally low in Finnish lakes (Conde and Sanz Alaejos 1997), which decreases the likelihood of its toxicity and protective effect.

The placenta is a temporary organ existing only during gestation, connecting the foetus to the mother’s body. As such, it plays a central role in nutrition, respiration, and waste removal, which occur only via placental transfer. Therefore, potentially detrimental elements, such as mercury, or beneficial elements, such as selenium, are also transferred to the foetus through the placenta (Grajewska et al. 2020). Chemicals have been observed in placentas and pup tissues after parturition (Ask et al. 2002; Gundacker et al. 2010; Al-Saleh et al. 2011; Nehring et al. 2017). Nehring et al. (2017) proposed that the transfer of mercury into the placenta and the foetus could reduce the maternal body burden of mercury. Based on the mass balance calculations of Lyytikäinen et al. (2015), Saimaa ringed seal mothers allocate ca. 11% of their mercury burden to their pups during gestation (ca. 1%) and lactation (ca. 10%). In most mammals, mothers eat the placenta after parturition (Kristal et al. 2012) and, therefore, placentas are generally absent from wildlife toxicity studies. Phocids are one exception to this rule (Robinson and Pomeroy 2022). Consequently, Saimaa ringed seal placentas can be found in the vicinity of birth lairs situated on the shorelines of islets. So far, placentas have been used in population monitoring and genetic studies (Auttila et al. 2014; Valtonen et al. 2015), but in the hereby study, are used to investigate a contaminant burden in the population.

The main goals of our study are (1) to determine the current mercury concentrations from Saimaa ringed seal lanugo pup tissues (blubber, brain, kidney, liver, muscle, and placenta), (2) to evaluate the potential toxicity of mercury to seals by comparing observed concentrations in brain and liver tissues to thresholds set in the literature, and lastly, (3) to study the suitability of the placenta as a biomonitoring tool for mercury exposure.

## Materials and methods

### Sampling

**Fig. 1:**
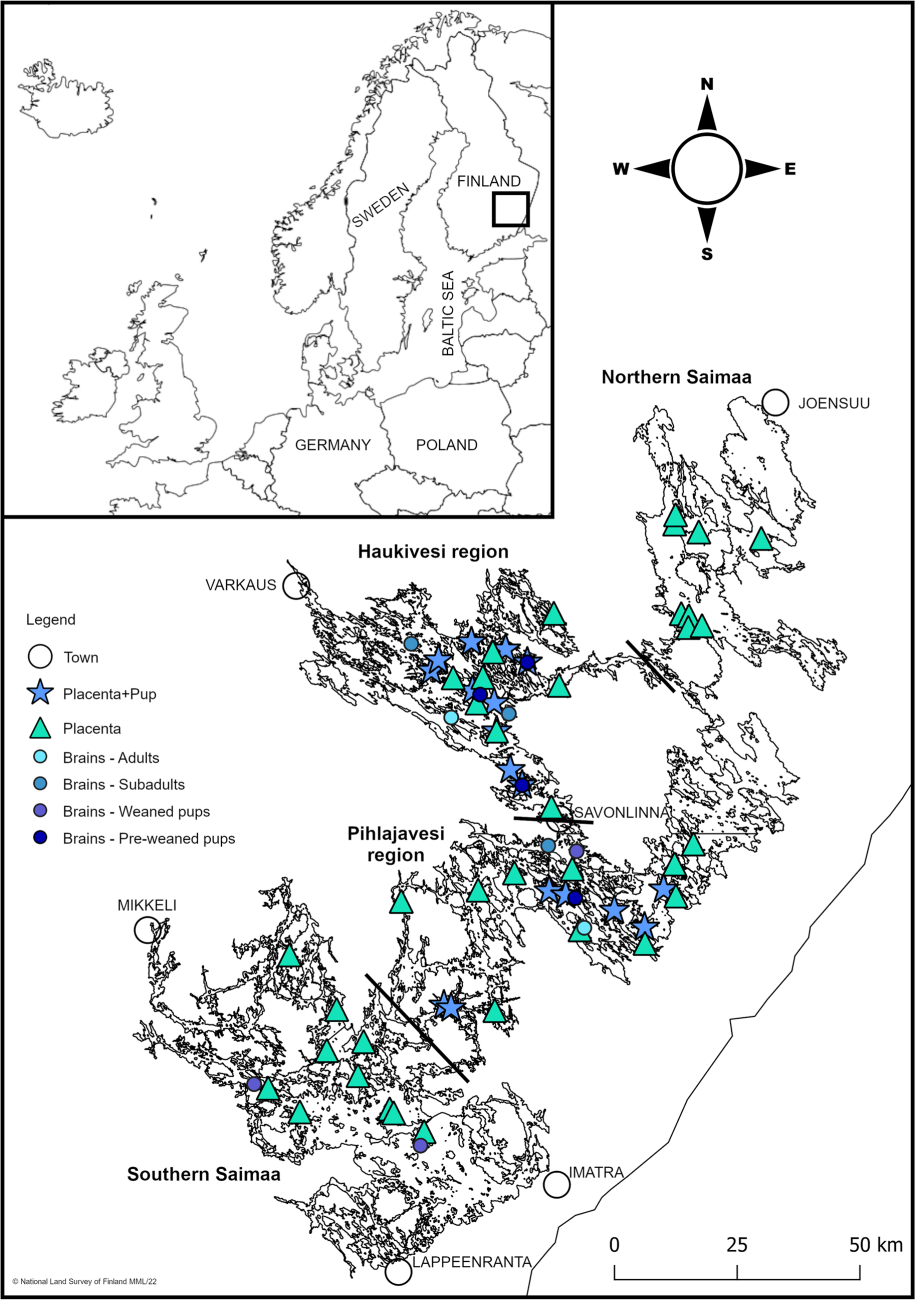
Map of Lake Saimaa and sampling sites for tissues of ringed seals. Black lines separate the four defined study regions

Seal tissue samples were collected between 2014 and 2023 from Lake Saimaa (61° 05′ to 62° 36′ N, 27° 15′ to 30° 00′ E), which is the largest lake in Finland (Fig. 1). Placentas were collected from the vicinity of birth lairs during the annual lair censuses or by diving immediately after ice break-up (Auttila et al. 2014). Additionally, dead seals of varying ages were gathered throughout the lake. Some of these seals were by-caught by gillnets and were fresh, while others were highly decomposed. Several tissue samples were collected (blubber, kidney, liver, and muscle) during post-mortem examinations, along with skulls (with intact brains), when available. The samples were stored at − 20 °C in the Saimaa ringed seal tissue bank maintained by the University of Eastern Finland (UEF) at the Joensuu campus. Sample collection was authorised by the Centre for Economic Development, Transport and the Environment (permit number VARELY/3480/2016).

**Fig. 2:**
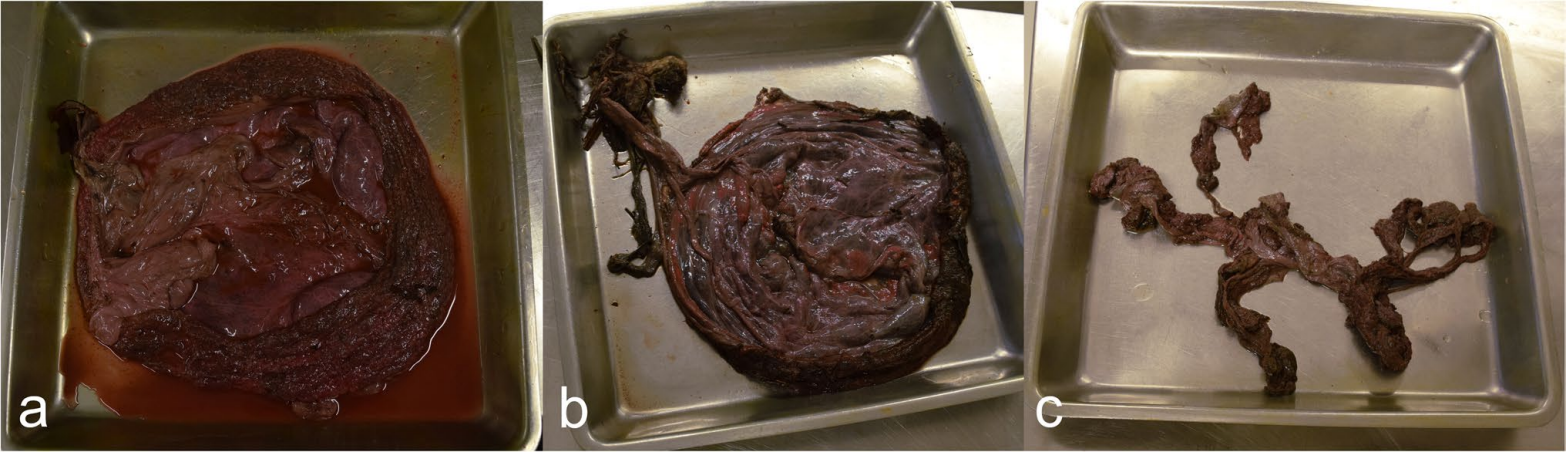
Placentas of Saimaa ringed seals in different conditions: **a** good, **b** average, and **c** poor

In our study, Lake Saimaa was divided into four main water basins: northern Saimaa, Haukivesi, Pihlajavesi, and southern Saimaa, to compare regional differences in tissue concentrations (Fig. 1). A total of 55 placentas were sampled and assigned to one of the three condition groups based on their decay state. Visual assessment was based on colouring and texture of the placenta: (a) good, with pinkish colour, intact texture, and fresh smell; (b) average, pinkish colouring has already begun fading, and grey/brown can be seen, texture shows signs of decay accompanied with a bad odour; and (c) poor, with clear signs of tissue decay together with a rancid odour and grey/brown colouring (see Fig. 2). Most placentas had been rinsed clean during collection, but five were given an additional gentle rinsing under running tap water to remove any dirt, so that external materials would not confound the chemical analyses.

Of the sampled placentas, 17 specimens could be connected to a dead pup. These pups were either found with the placenta still attached via the umbilical cord or the placenta was found in close vicinity of the pup carcass by divers. Five pups were stillbirth, ten died shortly after birth in the lair (one was killed by a red fox (*Vulpes vulpes*)), and two were older but pre-weaned (less than 3 months old). These pups had all or some of their lanugo hair (hair grown in utero), which is usually fully moulted when the pups are approximately 3 months old (Kunnasranta et al. 2021). These 17 pups are henceforth referred to as lanugo pups. The pups that were found dead in lairs showed evidence of milk consumption, i.e. milk was found in their stomachs and/or they had a visible layer of blubber.

Kidney, liver, and muscle samples were collected from these 17 pups, along with blubber (n = 3) and brain (n = 2) samples when available (Online resource 1 Table 1). We extracted additional ten brains from the skulls of seals from various age classes: two pre-weaned pups, three weaned pups (3–12 months), three subadults (1–3 years old), and two adults (> 3 years old) (Online resource 1 Table 2). These individuals, from the year 2015 to 2021, were found dead from different regions of the lake. They had died either by natural causes or by drowning in gillnets. A whole brain sample was collected from a skull with a plastic spoon through the occipital foramen to preserve the skull intact for future research. For chemical analyses, we only selected brains that still had some visible texture, bright pinkish colouring, and ones that did not have a strong smell of decay.

Sample selection, brain extractions, and preparation for shipping were performed at the UEF facilities on Joensuu campus, Finland. Containers and spoons used for sampling were acid-washed for at least 24 h, and we used sterile, single-use blades. All tissues were sent frozen for chemical analyses, on four separate occasions between mid-2021 and early 2024.

### Chemical analyses

Chemical analyses were performed by the ALS Scandinavia AB laboratory in Luleå, Sweden. Concentration determinations were performed for THg, MeHg, and total selenium (TSe). THg was analysed from all samples, whereas MeHg and TSe were analysed from 42 placentas and from five lanugo pups. THg and TSe samples were prepared by microwave-assisted acid digestion, and the analysis was performed with ICP-SFMS in accordance with Engström et al. (2004). A set of control blanks were prepared for both chemicals. Results for these blanks were 0.9, 0.9, 1.2, 0.4, and 0.3 ng/g for THg and 0, 0, 2, 3, and 3 ng/g for TSe. In-house quality control was performed with Spirulina powder, with concentrations calibrated against NIST SMR 1547. The results for the quality control were 9.0, 9.1, 10.1, 10.4, and 9.6 ng/g for THg (target 8, range 4–13 ng/g) and 153, 159, 182, 192, and 147 ng/g for TSe (target 168, range 82–245 ng/g). MeHg was analysed with GC-ICPMS. The tissue samples were initially spiked with enriched methyl ^198^Hg standard, after which potassium hydroxide in methanol was added. Next, MeHg was leached, and the samples were analysed. MeHg released from the sample was converted into ethylmethylmercury by aqueous phase ethylation. Ethylmethylmercury was driven out of solution, which was then captured on sorbent polymer. The ethylated MeHg was transferred to a GC-ICPMS instrument by heating the sorbent polymer. During this process, a porous polymer was separated from the mercury compounds, and the concentration was then determined using a species-specific isotope dilution method. For quality control, blanks (*n* = 3, 0.08, 0.07, 0.03 ng/g) and quality control solutions were prepared with the samples. For reference material, NIST SRM 2974a with a MeHg concentration of 69.06 ± 0.81 ng/g was used. The results were 68.0, 73.5, and 86.5 ng/g for the SRM2974a reference material (target 69.1, range 48–90 ng/g).

Additionally, to perform dry weight adjustment from the wet weight results, a piece from each of the sampled tissue was dried in an oven (Memmert model 400) at the UEF laboratory, following a protocol adapted from Gardner et al. (1985). The crucible cups used to hold the samples were dried overnight at 105 °C and weighed with a microscale (Denver Instrument SI-234). After sampling, the cups with tissues were weighed before being placed in an oven. Samples were then dried for 2 nights at 105 °C and weighed after both nights. Three placentas were omitted from the drying due to insufficient tissue material.

### Toxicity evaluation

For the mercury toxicity evaluation, we used a marine mammal liver threshold of < 16 µg/g ww. from Dietz et al. (2021), which is based on the study results of Ronald et al. (1977) and a mammalian brain threshold of < 0.1 µg/g ww. described by Krey et al. (2015), which is based on a literature review conducted by the authors (Table 1).

**Table 1:** The literature threshold levels used for evaluating mercury toxicity for mammals

Threshold	Level	THg Conc. (ww.)	Example of effects
Marine mammal liver (Dietz et al. 2021)	No risk	< 16 µg/g	The likelihood for adverse effects, such as effects on reproduction, physiology, condition, and behaviour, increases with an increase in risk level
	Low risk	16–64 µg/g	
	Moderate risk	64–83 µg/g	
	High risk	83–123 µg/g	
	Severe risk	≥ 123 µg/g	
Mammal brain (Krey et al. 2015)	Neurobehavioural changes	> 0.1 µg/g	Upregulation of genes and changes in locomotor activities
	Neurochemical changes	> 0.4 µg/g	Disruptions in receptor binding or enzyme activity in the brain tissue
	Neuropathological signs	> 4 µg/g	Loss of granule cells and neurons
	Clinical symptoms	> 6.75 µg/g	Gait disorders, clonic convulsions, vomiting, blindness, recumbency, and finally death

### Statistical analyses

Se:Hg molar ratio was calculated based on molar weight (Dehn et al. 2005; Cáceres-Saez et al. 2013) (Online resource 1 Eq. 1). The percentage of MeHg out of THg (%MeHg) was calculated for all tissues (Dietz et al. 2021) (Online resource 1 Eq. 2). In 13 samples (2 placenta, 2 muscle, 2 blubber, and 7 brain) the %MeHg was over 100%. This could imply an inconsistency in the laboratory analysis of MeHg. For this reason, we subsequently focus only on THg and TSe concentrations, and on Se:Hg molar ratios, while the results for MeHg and %MeHg are presented in the supplementary information. Dry weight–adjusted data were calculated by multiplying the wet weight concentration by the 2-night drying percentage. Three placentas did not have enough tissue left for drying, which is why the wet weight and dry weight–adjusted datasets had different sample sizes. To counter this difference in sample sizes, we performed three sets of statistical analyses: one with the wet weight samples, one with the dry weight–adjusted samples, and one where the three placentas that could not be dried were removed from the wet weight dataset to match the number of dry weight–adjusted samples.

For statistical analysis, Shapiro–Wilk’s test was used to test the normality of model residuals and base-10 logarithmic transformation was applied where normality was not met. The homogeneity of variances of the model residuals was tested with Levene’s test, while Rosner’s test was used to check the model residuals for possible outliers. The Tukey HSD test was used as a post hoc test for one-way and two-way ANOVA analysis. Two-way ANOVA was used to investigate whether milk consumption influenced THg concentrations in pups. Out of the 17 pup-placenta pairs which were analysed for THg, four individuals had not consumed milk. Other chemicals were analysed from only five pup-placenta pairs and thus were omitted from this analysis, as only one pup out of the five sampled placenta-pup pairs had not consumed milk. Two-way ANOVA was also used to study the possible effect of region and placental condition on the chemical concentrations. We used the Scheirer-Ray-Hare test to study the effect of sex and tissue type on the THg concentrations in lanugo pups, as the data requirements for two-way ANOVA were not met. Because we only had concentration data for MeHg and TSe from three females and two males, these two chemicals were omitted from statistical analysis. One-way ANOVA was used to test the differences of placental conditions within regions, the differences in chemical concentrations between regions for the good-quality placentas, and the differences in chemical concentrations among pup tissues. Additionally, one-way ANOVA was used to study the possible variation in drying percentage according to placental condition. The Kruskal–Wallis test was used when residuals were not normally distributed even after logarithmic transformation. The Dunnett test was used as a post hoc test for Kruskal–Wallis tests.

To further investigate the possible differences between regions and placental conditions, a factor analysis of mixed data (FAMD) was performed for placentas with THg concentrations, TSe concentrations, and Se:Hg molar ratios (all placentas *n* = 42, dry weight–adjusted *n* = 39, wet weight with dry weights *n* = 39). Because of the inconsistency in MeHg and %MeHg laboratory analysis, FAMD was performed without the MeHg and %MeHg data. The variance percentage explained by each dimension was inspected using eigenvalues and a scree plot. Both qualitative and quantitative variables were inspected separately using correlation circles. Finally, a factor map for FAMD was generated to obtain an overview of the results of the two most impactful dimensions for regions and placental conditions.

We performed correlation tests between the placentas that could be connected to individual pups and pup tissues. The concentration data were tested for normality with Shapiro–Wilk’s test. From the correlation matrix, the normally distributed and statistically significant (*p* < 0.05) correlations were further inspected with regression analysis. Data linearity was visually inspected and model residuals were tested for normality with Shapiro–Wilk’s test. Homoscedasticity was tested with the Breusch-Pagan test. Finally, we visually evaluated the model fit using a regression line. All statistical analyses were carried out using R Statistical Software (v4.4.0; R Core Team 2024).

## Results

All analysed chemicals were detected throughout the samples. In the lanugo pups, the highest concentrations of THg and TSe were found in the livers and kidneys, whereas the highest Se:Hg molar ratios were observed in the placentas (Fig. 4, Online resource 1: Table 7 and Table 8). The Se:Hg molar ratio was above the threshold ratio of 1:1 in most analysed samples (84.6% of all samples with Se:Hg molar ratio available, *n* = 65). The lowest Se:Hg molar ratios were observed in brains, as most samples had ratios below 1 (70% of the analysed brain samples, *n* = 12).

### Placentas

Placentas contain THg (mean 103 ng/g ww.), TSe (mean 266 ng/g ww.), and MeHg (mean 50 ng/g ww.) (Table 2, Online resource 1: Table 3 and Table 4). In general, placentas contained higher concentrations of TSe (range 100–527 ng/g ww.) than THg (range 12–299 ng/g ww.). This is further highlighted by the fact that the Se:Hg molar ratios are above 1 in all but one sample. Out of the sampled placentas, 46% were in good, 38% in average, and 16% in poor condition.

**Table 2:** THg and TSe concentrations (ng/g ww.) and Se:Hg molar ratios in Saimaa ringed seal placenta samples

Chemical	*n*	Mean	Median	St.dev	Min	Max
THg	55	103	85	65	12	299
TSe	42	266	229	127	100	527
Se:Hg	42	10.2	8.5	8.1	0.8	38.3

We found no interaction between the region from which the placenta was collected and the condition of the placenta regarding the chemical concentrations in the placentas (Online resource 2). However, THg concentrations were higher in good-condition placentas (mean 131 ng/g ww., *n* = 24) compared to those in poor condition (mean 72 ng/g ww., *n* = 9) (*p* = 0.02, *n* = 55). This effect was not statistically significant if the concentrations were calculated by dry weight–adjusted placentas (*n* = 52). Close inspection reveals that the difference we observed between placenta conditions when all placentas are considered (*n* = 55) stems from the Pihlajavesi region, where the good-condition placentas (mean 123 ng/g ww., *n* = 5) had higher THg concentrations than did those in poor condition (mean 29 ng/g ww., *n* = 3) (*p* = 0.008, *n* = 17) (Online resource 2). No other region showed such a statistical difference. This effect was also present in the dry weight–adjusted data (*p* = 0.02, *n* = 16) and in the wet weight placentas with dry weight data (*p* = 0.02, *n* = 16). The southern Saimaa region had higher placental concentrations of THg (mean 107 ng/g dw., *n* = 10) than the Pihlajavesi region did (mean 64 ng/g dw., *n* = 17), but this effect was only seen in dry weight–adjusted data (*p* = 0.04, *n* = 52). When all placentas were considered, Pihlajavesi region (mean 16.7, *n* = 10) had a higher Se:Hg molar ratio than did northern Saimaa (mean 6.1, *n* = 9) (*p* = 0.02, *n* = 41), but this effect was not present in the dry weight–adjusted data. We observed no regional differences in the analysed chemicals when only good-condition placentas were used (*n* = 25) (Online resource 2).

**Fig. 3:**
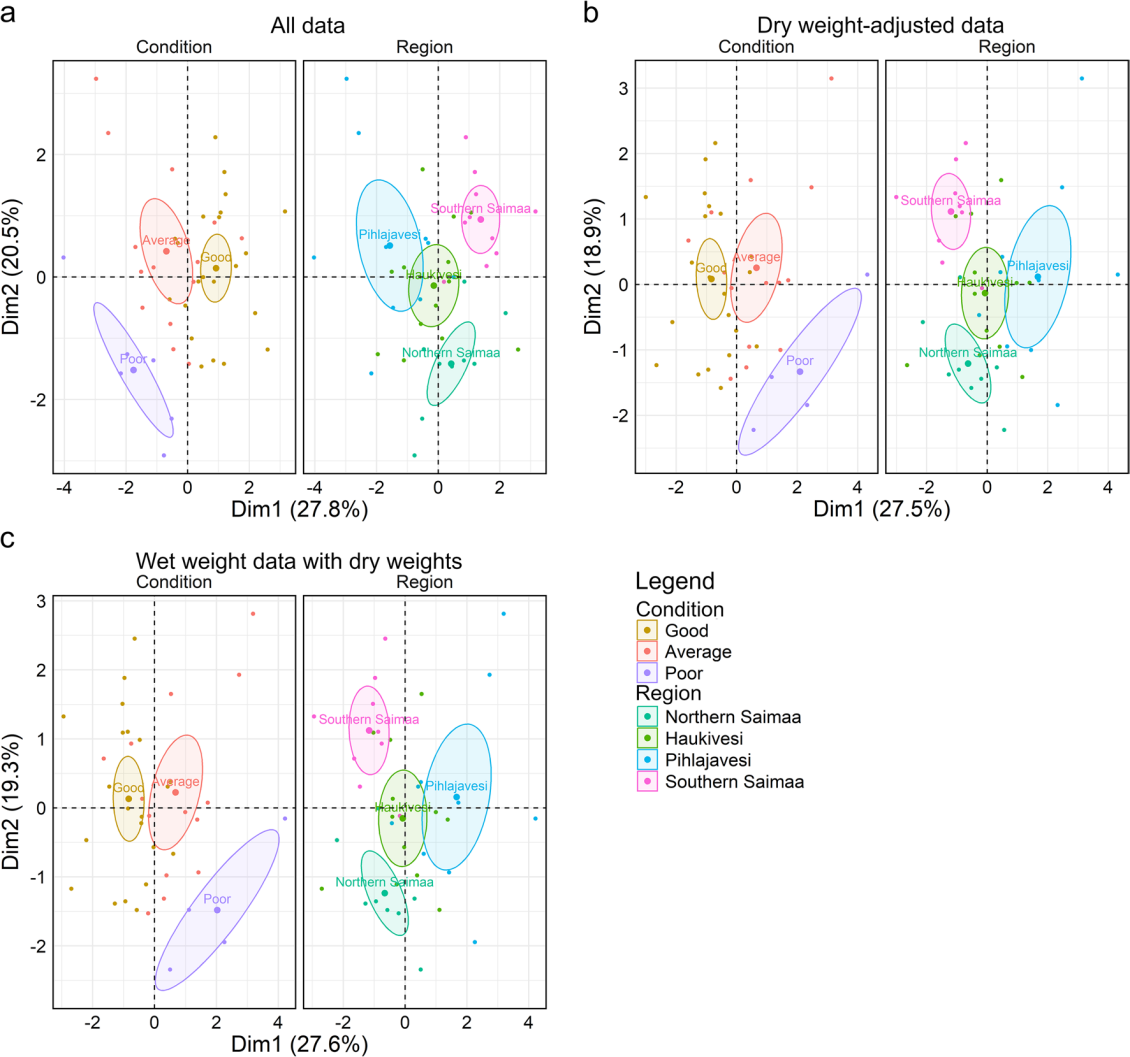
FAMD factor maps for Saimaa ringed seal placentas, including region, condition, total mercury (THg), total selenium (TSe), and selenium to mercury (Se:Hg) molar ratio data. Map **a** has all placentas (*n* = 42) and maps **b** and **c** contain dry weight–adjusted data (*n* = 39) and wet weight data with dry weights (*n* = 39), respectively. The maps show the first two dimensions and are divided by the qualitative variables “condition” and “region”. Ellipses show 0.95 confidence around the group mean points

The FAMD analysis provided further support for the differences between geographic regions and sample conditions found in the ANOVA and post hoc tests, when a combination of THg, TSe, and Se:Hg molar ratio was considered (Fig. 3, Online resource 1: Table 5 and Table 6). Although there were differences between all the condition classes, placentas in poor condition differed the most from placentas in good and average conditions. This was evident regardless of whether all placentas (*n* = 42) (Fig. 3a), dry weight–adjusted (*n* = 39) (Fig. 3b), or wet weight placentas with dry weight (*n* = 39) (Fig. 3c) were included in the analysis. Concerning the regional differences, the largest distinctions were found between Northern and Southern Saimaa and Northern Saimaa and Pihlajavesi basin, while the least differentiations were between the central areas of the lake (Haukivesi and Pihlajavesi basins) (Fig. 3a). Also, these regional differences persisted when including only dry weight (Fig. 3b) or wet weight samples (Fig. 3c).

### Lanugo pups

When all lanugo pup tissues (total *n* of tissues = 72) were considered, THg concentrations were affected by the interaction between tissue type and milk consumption (*p* = 0.04) (Online resource 2). However, no statistically significant differences were found within the tissue type between the individuals that had consumed milk and those that had not. Other observed interactions between tissue types and milk consumption were not biologically meaningful. Dry weight–adjusted data (*n* = 70) and wet weight data with dry weights (*n* = 70) showed no difference in tissue THg concentrations between pups that drank milk and those that did not. Similarly, the sex of the pup had no effect on tissue THg concentrations (Online resource 2). However, lanugo pups from the Haukivesi region had higher tissue THg concentrations than did lanugo pups from the Pihlajavesi region (Online resource 2), although this difference was only seen in the wet weight data with dry weight (*p* = 0.04, *n* = 66).

**Fig. 4:**
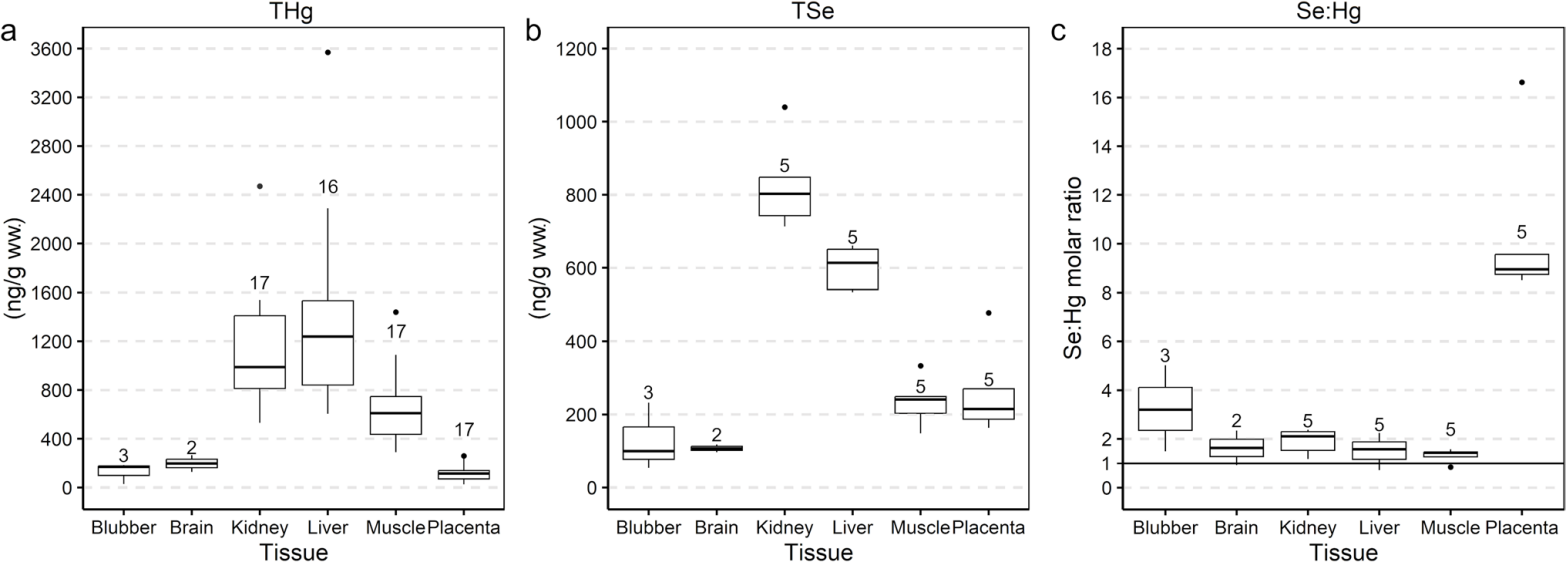
Total mercury (THg, **a**) and total selenium (TSe, **b**) concentrations and selenium to mercury (Se:Hg) molar ratios (**c**) in Saimaa ringed seal lanugo pups (*n* = 17) (Online resource 1 Table 7). The numbers above boxes denote the number of samples for each tissue. The solid lines within the boxes represent the medians, the boxes cover the middle 50% of the data, the whiskers represent the minimum and maximum values excluding data points that the boxplot has deemed to be outliers, which are shown as dots. Solid line in Se:Hg molar ratios (**c**) denotes the 1:1 threshold ratio

The highest THg concentrations were found in lanugo pup kidneys (mean 1142 ng/g ww., *n* = 17) and livers (mean 1373 ng/g ww., *n* = 16) (Online resource 1: Table 7 and Table 8). These levels were significantly higher than in the other analysed tissues (Fig. 4, Table 3, and Online resource 2). Similar patterns were observed in the dry weight–adjusted and wet weight data with dry weights data sets (Online resource 2). The highest TSe concentrations were also observed in the kidneys (mean 829 ng/g ww., *n* = 5) and livers (mean 600 ng/g ww., *n* = 5) and were significantly different from the other tissues (Table 3). As with THg, the TSe results were the same for dry weight–adjusted and wet weight data with dry weight datasets. The highest Se:Hg molar ratios were detected in the placentas associated with lanugo pups (mean 10.4, *n* = 5) and were significantly different from the ratios from the other tissues (blubber *p* =  < 0.01, brain *p* =  < 0.01, kidney *p* < 0.01, liver *p* < 0.01, muscle *p* =  < 0.01). The differences in placental Se:Hg molar ratios were also observed in the dry weight–adjusted and in the wet weight data with dry weight datasets.

**Table 3:** *p*-values of a post hoc test for one-way ANOVA analysis exploring the differences between lanugo pup tissue types and their concentrations of total mercury (THg) and total selenium (TSe) concentrations (ww. *n* = 72). Statistically significant results are in bold. One-way ANOVA *F*-statistic 59.58 and degrees of freedom 5

THg	Blubber	Brain	Kidney	Liver	Muscle	Placenta
TSe						
Blubber		0.71	** < 0.01**	** < 0.01**	** < 0.01**	1.00
Brain	1.00		** < 0.01**	** < 0.01**	**0.03**	0.58
Kidney	** < 0.01**	** < 0.01**		1.00	**0.02**	** < 0.01**
Liver	** < 0.01**	** < 0.01**	0.70		** < 0.01**	** < 0.01**
Muscle	0.08	0.15	** < 0.01**	** < 0.01**		** < 0.01**
Placenta	**0.05**	0.09	** < 0.01**	** < 0.01**	1.00	

For THg concent﻿ration, we observed correlation between lanugo pup placentas and livers (cor = 0.5, *p* = 0.03), as well as placentas and muscles (cor = 0.5, *p* = 0.03) (Online resource 2), which were further supported by the regression analysis (liver *r*^2^ = 0.29, *p* = 0.03, *n* = 33, muscle *r*^2^ = 0.28, *p* = 0.03, *n* = 34) (Online resource 2). However, these results were only seen when all data were considered.

### Mercury concentrations in the brain

**Fig. 5:**
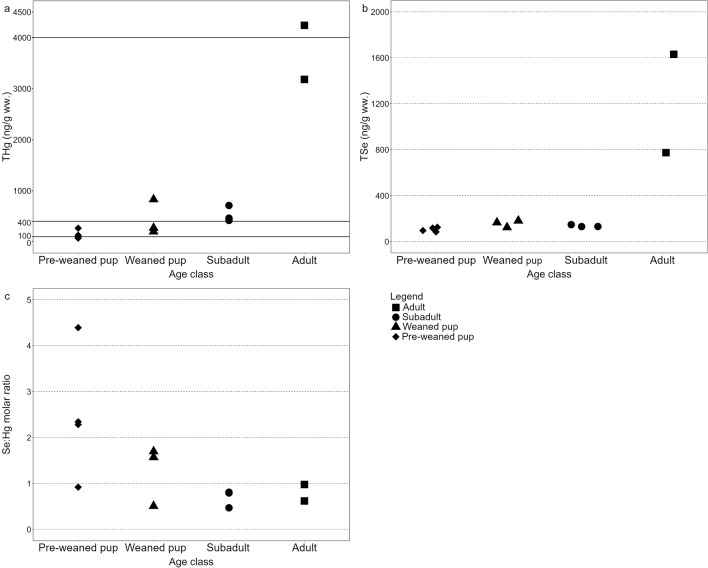
Concentrations of total mercury (THg, ng/g ww.) in the brains of Saimaa ringed seals by age class (*n* = 12). Solid lines indicate the three lowest thresholds from Krey et al. (2015); neurobehavioural changes (100 ng/g ww.), neurochemical changes (400 ng/g ww.), and neuropathological signs (4000 ng/g ww.)

As with the other tissues, all the analysed chemicals were present in Saimaa ringed seal brains (Online resource 1: Table 9 and Table 10). Adults had the highest brain THg concentrations (mean 3710 ng/g ww., *n* = 2) while pre-weaned pups had the lowest levels (mean 140 ng/g ww., *n* = 4) (Fig. 5a). Brain THg concentrations in adult seals were significantly higher than in any other age class (subadults *p* = 0.02, weaned pups *p* = 0.006, pre-weaned pups *p* = 0.0004, *n* = 12), but the difference for TSe was only found between adult seals and pre-weaned pups (*p* = 0.03, *n* = 12) (Fig. 5b and Online resource 2). In addition, subadults had significantly higher brain THg concentrations than pre-weaned pups (*p* = 0.03, *n* = 12). In the case of Se:Hg molar ratios, the older seals, adults (range 0.6–1.0, *n* = 2), and subadults (range 0.5–0.8, *n* = 3) were below the threshold ratio of 1:1, whereas only one weaned (range 0.5–1.7, *n* = 3) and one pre-weaned pup (0.9–4.4, *n* = 4) were below the threshold ratio of 1:1 (Fig. 5c). Generally, brain THg concentrations increased with seal age, whereas the opposite was true for Se:Hg molar ratios. In the dry weight–adjusted dataset, adult seal brains had higher THg concentration than did the brains of the younger age classes (subadults *p* = 0.0122, weaned pups *p* = 0.0057, pre-weaned pups *p* = 0.0004).

## Discussion

Our study indicates that Saimaa ringed seals are still exposed to mercury. We observed a similar level of mercury contamination in lanugo pup livers and muscle tissues than levels reported from 2002 to 2012 by Lyytikäinen et al. (2015). Moreover, liver selenium concentrations were at the same level as at the beginning of the 2000s, but levels were lower in muscle tissues. However, in our study, selenium was only analysed from five lanugo pups. When compared to other pinniped populations, Saimaa ringed seal pups have higher contaminant levels than ringed seal (*P. h. hispida*) pups in Canada (Smith and Armstrong 1978), relatively similar levels to those in Greenland (AMAP 2002), but lower levels than in Pacific harbour seal (*Phoca vitulina richardii*) pups in the USA (Brookens et al. 2008). We also observed an accumulation of mercury in Saimaa ringed seal brains. The mercury concentrations in the brains exceeded the threshold levels described by Krey et al. (2015) which could lead to potential neurological malfunctions.

### Toxicity evaluation

All lanugo pup liver mercury concentrations (range 604–3570 ng/g ww., *n* = 16) from Saimaa ringed seals were below the threshold (< 160,000 ng/g) characterised to pose no risk for adverse health effects. However, despite detoxification mechanisms (López-Berenguer et al. 2020), mercury accumulates in seal brains and the contaminant concentration reaches concerning levels, especially in adults. Mercury accumulation in the brain and with age are in accordance with observations from other pinniped studies (e.g. Brookens et al. 2008; Krey et al. 2015; Desforges et al. 2021). Regardless of the higher mercury concentrations in the brains of subadult and adult seals, the pre-weaned and weaned pups may be more susceptible to adverse effects from mercury since their central nervous system is still developing (López-Berenguer et al. 2020). The brain concentrations of mercury exceeded the threshold for neurobehavioral changes (> 100 ng/g ww.) in two pre-weaned and all weaned pups in our study (Krey et al. 2015). In one weaned pup, the concentration even exceeded the threshold for neurochemical changes (> 400 ng/g ww.). The mercury concentrations in the brains of all adults and subadults surpassed the threshold for neurochemical changes, while the mercury concentration of one adult even exceeded the threshold for neuropathological signs (> 4000 ng/g ww.).

The Se:Hg molar ratios are another significant aspect of the mercury toxicity evaluation. For lanugo pups, the liver Se:Hg molar ratio was available for five individuals, with one having a ratio below the threshold of 1:1. This was the individual with the highest liver mercury concentration. Additionally, one of the pre-weaned and one of the weaned pups had brain Se:Hg molar ratios below the threshold of 1:1. Mercury could therefore potentially cause adverse effects in Saimaa ringed seal pups in severe cases, especially in the older pups. These effects could manifest, for example, as changes in locomotion or as alterations in brain chemistry (Krey et al. 2015).

In all subadult and adult seals, the brain Se:Hg molar ratio was below the threshold of 1:1. Curiously, the adult seal with the most severe case of mercury contamination (4240 ng/g ww.) had a brain Se:Hg molar ratio of 0.98. Despite this, the overall Se:Hg molar ratios in older seals were not high enough to protect them from high brain mercury concentrations and thus, adverse effects are possible such as changes in locomotion, alterations in brain chemistry, and even loss of neurons (Krey et al. 2015). The number of brain tissue samples in our study was low, and hence, the toxicity evaluation should be treated with caution.

We were not able to establish any correlation between the stillbirths and the mercury concentrations. However, individual seals in both the lanugo and weaned pup groups had elevated mercury concentrations in their brain and had Se:Hg molar ratios below the threshold level of 1:1. Lyytikäinen et al. (2015) observed a mean mercury concentration of 69,000 ng/g ww. (range 12,000–246,000 ng/g ww.) in the livers of adult Saimaa ringed seals, exceeding the threshold for moderate risk for adverse health effects for marine mammals (Table 1), affecting, for example, reproduction. In the same study, the highest mercury concentration observed in the liver (246,000 ng/g ww.) exceeds the threshold for high risk for adverse health effects. Thus, mercury concentrations in adult seals could affect their reproduction, and therefore, mercury could have a partial contribution to the stillbirths observed in the population. Other contributors may include low selenium concentrations (Hosnedlova et al. 2017), other chemicals (Dagher et al. 2020), and the combined effects of a mixture of chemicals.

As observed in previous studies on Saimaa ringed seals (Kari and Kauranen 1978; Hyvärinen and Sipilä 1984; Lyytikäinen et al. 2015), mercury concentrations have decreased during the past few decades, which may be due to legislative efforts to decrease mercury emissions (e.g. the ban of mercury in pulp industry in the 1970s and Regulation 2017/852 of the European Parliament and of the Council). Despite the obvious decrease in tissue mercury concentrations in Saimaa ringed seals, results from our study and in Siimes et al. (2019) indicate that mercury could still accumulate into seals and other biota in Lake Saimaa at relatively high concentrations. The persistence of mercury contamination may be due to legacy mercury pollution from industrial sources, atmospheric transport, leaching from surrounding catchment areas (especially peatlands), and from urban areas surrounding the lake (Verta 1990). Due to legacy contamination and multiple possible current contamination sources, the future of mercury pollution at Lake Saimaa is hard to predict and thus regular monitoring is advised.

### Placenta in biomonitoring

All placentas used in our study contained mercury. Moreover, as mercury was observed in all stillborn pups, the placenta does not seem to prevent its transfer into the foetus during gestation. These findings are in accordance with earlier findings from other mammals such as Baltic grey seals (*Halichoerus grypus grypus*) and humans (Ask et al. 2002; Al-Saleh et al. 2011; Nehring et al. 2017; Tong et al. 2021). We additionally found that the mercury level present in the placenta correlates with the concentrations observed in other tissues. Thus, the placenta could be used as a non-invasive tool to biomonitor chemical exposure levels in the Saimaa ringed seal population.

Placentas can be collected from lair sites in spring, after ice break-up, without disturbing the seals. Placentas are, generally, in better condition than seal carcasses. The lake’s cold water during the pupping season preserves the placentas, which are mostly found in average to good condition. Thus, most of the collected placentas are suitable for the approach described in the presented study, and our data could serve as a baseline for future monitoring. However, it is noteworthy that, based on our study, regional differences in placental mercury concentrations may exist within Lake Saimaa. As, Saimaa ringed seals show strong site fidelity (Koivuniemi et al. 2016; Biard et al. 2022; but see Niemi 2013), the observed regional differences are likely connected to the local environmental conditions. These differences may be due to variations in human population density and industry around the lake or in environmental factors driving methylation of mercury into methylmercury.

The chemical analyses in our study were conducted on a wet weight basis, which could increase the variation in mercury concentrations. This effect should be studied further, as our dry weight–adjusted data did not always yield similar results as the wet weight data. Nonetheless, the placental tissue proved itself to be an ideal sample source for the non-invasive monitoring of mercury concentration in a free-living population.

## Conclusions

Despite the decrease in mercury exposure during past decades, the concentrations in Saimaa ringed seals remain relatively high. Based on the toxicity evaluation, the pups of this population may suffer, in severe cases, from adverse effects caused by mercury toxicity, although the bioaccumulation of mercury and low selenium concentrations in older seals indicate that adverse effects from mercury toxicity are possible and are more likely in older than in younger seals. Mercury concentrations in seal pups could not be connected to stillbirths, but a partial contribution may be possible. In conclusion, regular mercury concentration monitoring is relevant for this endangered population, and the placenta may potentially serve as a valuable non-invasive monitoring tool.

## Supplementary Information

Below is the link to the electronic supplementary material.

Supplementary file1 Additional experimental information includes the equations for Se:Hg molar ratio and %MeHg, detailed information of the sampled tissues per placenta-connected lanugo pup, and age groups for brain samples in online resource 1 (PDF 226 KB)

Supplementary file2 Additionally detailed information of the statistical analysis results are given in online resource 2 (XLSX 61 KB)

Supplementary file3 Furthermore, the individual tissue chemical analysis data are also available in online resource 3 (XLSX 32 KB)

## Data Availability

Data will be available as online resource.
