# Correction to: Mercury exposure in ringed seals (*Pusa hispida saimensis*) in Lake Saimaa, Finland, and the placenta as a possible non‑invasive biomonitoring tool

**DOI:** 10.1007/s11356-024-35348-6

**Published:** 2024-10-18

**Authors:** Jesse Simola, Mervi Kunnasranta, Marja Niemi, Vincent Biard, Jarkko Akkanen

**Affiliations:** 1 Department of Environmental and Biological Sciences, University of Eastern Finland, P.O. Box 111, FIN‑80101 Joensuu, Finland; 2 Natural Resource Institute Finland, Yliopistokatu 6, FIN-80100 Joensuu, Finland

**Correction to: Environmental Science and Pollution Research (2024) 31:57720–57732**

10.1007/s11356-024-34980-6

The Authors unfortunately forgot to acknowledge one of our partners in the “Acknowledgements section”.

The text “We would like to acknowledge that the field collection of samples was performed by employees of Metsähallitus, Parks and Wildlife Finland. We would like to thank Mairi Young and Milaja Nykänen for their help in finalising the manuscript.” should be included in the Acknowledgements text.

The Original article has been corrected.

