# Supplementary material for: Mercury exposure in ringed seals (Pusa hispida saimensis) in Lake Saimaa, Finland, and the placenta as a possible non-invasive biomonitoring tool
Source: Environ Sci Pollut Res Int. 2024 Sep 18;31(47):57720–32. doi: 10.1007/s11356-024-34980-6 (PMC11467087; doi:10.1007/s11356-024-34980-6)
Supplement: Supplementary file 1 — Supplementary file1 Additional experimental information includes the equations for Se:Hg molar ratio and %MeHg, detailed information of the sampled tissues per placenta-connected lanugo pup, and age groups for brain samples in online resource 1 (PDF 226 KB) [file 11356_2024_34980_MOESM1_ESM.pdf]

Mercury exposure in ringed seals (Pusa hispida saimensis) in Lake Saimaa, Finland and the placenta as a possible non-invasive biomonitoring tool

Environmental Science and Pollution Research

Jesse Simola,[1*] Mervi Kunnasranta,[1,2] Marja Niemi,[1] Vincent Biard,[1] Jarkko Akkanen[1]

1) Department of Environmental and Biological Sciences, University of Eastern Finland, P.O. Box 111, FIN-80101, Joensuu, Finland

2) Natural Resource Institute Finland, Yliopistokatu 6, FIN-80100, Joensuu, Finland

* E-mail address of the corresponding author: jesse.simola@uef.fi

Table 1 Analyzed tissues of Saimaa ringed seal pups (n = 17). X denotes which tissues were available for these individuals.

Pup ID	Blubber	Brain	Kidney	Liver	Muscle	Placenta
2598			X	X	X	X
2599			X	X	X	X
2601			X	X	X	X
2604			X	X	X	X
2627			X		X	X
2632			X	X	X	X
2633			X	X	X	X
2669			X	X	X	X
2682			X	X	X	X
2708			X	X	X	X
2713			X	X	X	X
2758	X		X	X	X	X
2784	X	X	X	X	X	X
2786	X		X	X	X	X
2788		X	X	X	X	X

2790	X	X	X	X
2811	X	X	X	X

Table 2 The age groups used for Saimaa ringed seal brain sampling. Classification into an age group was based on the combined age determination from teeth, death records, and carcass body measurements (body length and weight).

Seal ID	Age group	Notes
2784	Pre-weaned pup	Could be connected to a placenta
2788	Pre-weaned pup	Could be connected to a placenta
2789	Pre-weaned pup	Could not be connected to a placenta
2799	Pre-weaned pup	Could not be connected to a placenta
2778	Weaned pup	
2806	Weaned pup	
2809	Weaned pup	
2754	Subadult	
2756	Subadult	
2782	Subadult	
2777	Adult	
2787	Adult	

Equation 1 Selenium to mercury molar ratio calculation.

Selenium to mercury (Se:Hg) molar ratio was calculated as follows (Dietz et al. 2000, Dehn et al. 2005, Cáceres-Saez et al 2012).

$$Se{:}Hg = \frac{(Se)/78.97}{(Hg)/200.59} \text{ (Equation 1)}$$

Where (Se) and (Hg) are total selenium and total mercury concentration within a tissue, respectively. 78.97 (National Center for Biotechnology Information 2021b) and 200.59 (National Center for Biotechnology Information 2021a) are the molecular weights of selenium and mercury, respectively.

Equation 2 Calculation for percentage of methylmercury out of total mercury.

Percentage of methylmercury (%MeHg) out of total mercury (THg) was calculated using the following equation (Dehn et al. 2005):

$$\%MeHg = \frac{(MeHg)}{(THg)} \times 100 \text{ (Equation 2)}$$

Where (MeHg) and (THg) are the concentration of methylmercury and total mercury within a tissue in ng/g ww., respectively.

Table 3 Methylmercury (MeHg) (ng/g ww.) and percentage of methylmercury out of total mercury (%MeHg) in Saimaa ringed seal placenta samples.

Chemical	n	Mean	Median	St.dev	Min	Max
MeHg	42	50	30	62	3	315
%MeHg	42	47	43	34	10	216

Table 4 Dry weight adjusted concentrations for total mercury (THg), total selenium (TSe), methylmercury (MeHg), percentage of methylmercury out of total mercury (%MeHg), and selenium to mercury (Se:Hg) molar ratio from Saimaa ringed seal placentas.

Chemical	n	Mean	Median	St.dev	Min	Max
THg	52	84	72	53	10	248
TSe	39	223	189	104	75	449
MeHg	39	42	25	53	3	261

%MeHg	39	39	36	30	7	185
Se:Hg	39	8.4	7.0	7.0	0.7	33.8

Table 5 Eigenvalues of the factor analysis of mixed data on placental variables: region of collection, condition, concentrations of total mercury, concentrations of total selenium, and selenium to mercury molar ratios.

Data set	Dimension	Eigenvalue	Variance %	Cumulative variance %
All data	1	2.2	27.8	27.8
	2	1.6	20.5	48.3
	3	1.0	13.1	61.4
	4	0.9	11.2	72.7
	5	0.8	9.7	82.4
Dry weight adjusted	1	2.2	27.5	27.5
	2	1.5	18.9	46.5
	3	1.1	13.6	60.1
	4	0.9	11.2	71.3
	5	0.8	10.4	81.7
Wet weight with dry weights	1	2.2	27.6	27.6
	2	1.5	19.3	46.8
	3	1.1	13.6	60.5
	4	0.9	11.2	71.7
	5	0.8	10.3	82.0

Table 6 Contribution of placental variables (region, condition, concentration of total mercury (THg), concentrations of total selenium (TSe), and selenium to mercury molar (Se:Hg) ratio) to the two first dimensions of the factor analysis of mixed data.

Data set	Variable	Contribution to dimension 1	Contribution to dimension 2
All data	THg	31.1	0.2
	TSe	1.6	41.6
	Se:Hg molar ratio	24.2	17.0
	Region	21.9	26.3
	Condition	21.1	14.8
Dry weight adjusted	THg	29.8	0.3
	TSe	0.9	48.4
	Se:Hg molar ratio	25.9	16.3
	Region	23.4	25.9
	Condition	20.1	9.1
Wet weight with dry weights	THg	29.7	0.5
	TSe	0.5	48.0
	Se:Hg molar ratio	27.2	14.9
	Region	22.9	26.0
	Condition	19.7	10.6

Table 7 Concentrations (ng/g) of total mercury (THg), concentrations of total selenium (TSe), concentrations of methylmercury (MeHg), percentage of methylmercury out of total mercury (%MeHg), and selenium to mercury (Se:Hg) molar ratio from the tissues of Saimaa ringed seal lanugo pups (n = 17) from years 2014-2022.

Chemical	Tissue	n	Mean	Median	St.dev	Min	Max
THg	Blubber	3	127	170	87	27	184
	Brain	2	197	197	98	128	266
	Kidney	17	1142	988	449	531	2470
	Liver	16	1373	1240	776	604	3570

	Muscle	17	651	611	292	289	1440
	Placenta	17	116	116	66	25	259
TSe	Blubber	3	129	100	92	54	232
	Brain	2	107	107	15	96	118
	Kidney	5	829	803	129	713	1040
	Liver	5	600	614	60	534	661
	Muscle	5	401	375	207	170	708
	Placenta	5	263	215	127	163	478
MeHg	Blubber	3	172	215	143	13	289
	Brain	2	198	198	103	125	270
	Kidney	5	383	323	190	238	713
	Liver	5	356	252	226	153	620
	Muscle	5	235	241	68	148	333
	Placenta	5	28	26	26	5	70
%MeHg	Blubber	3	111	117	62	46	170
	Brain	2	100	100	3	98	102
	Kidney	5	34	25	17	20	62
	Liver	5	32	27	18	12	59
	Muscle	5	89	94	35	29	116
	Placenta	5	37	50	19	13	52
Se:Hg	Blubber	3	3.2	3.2	1.8	1.5	5.0
	Brain	2	1.6	1.6	1.0	0.9	2.3
	Kidney	5	1.9	2.1	0.5	1.2	2.4
	Liver	5	1.5	1.6	0.6	0.7	2.2
	Muscle	5	1.3	1.4	0.3	0.8	1.6
	Placenta	5	10.5	9.0	3.5	8.5	16.6

Table 8 Dry weight adjusted data set concentrations (ng/g) of total mercury (THg), concentrations of total selenium (TSe), concentrations of methylmercury (MeHg), percentage of methylmercury out of total mercury (%MeHg), and selenium to mercury (Se:Hg) molar ratio from the tissues of Saimaa ringed seal lanugo pups (n = 17) from years 2014-2022.

Chemical	Tissue	n	Mean	Median	St.dev	Min	Max
THg	Blubber	3	9	10	7	1	15
	Brain	2	165	165	70	116	215
	Kidney	17	840	735	356	283	1897
	Liver	16	921	819	503	412	2272
	Muscle	17	455	402	209	194	1035
	Placenta	16	96	93	49	36	211
TSe	Blubber	3	8	8	6	2	13
	Brain	2	92	92	21	78	107
	Kidney	5	620	596	117	510	807
	Liver	5	401	378	56	348	491
	Muscle	5	163	166	49	97	228
	Placenta	4	233	196	106	153	387
MeHg	Blubber	3	12	12	12	0.5	25
	Brain	2	166	165	74	113	218
	Kidney	5	288	222	152	177	553
	Liver	5	228	178	128	106	397
	Muscle	5	281	259	151	116	497
	Placenta	5	28	23	21	8	57
%MeHg	Blubber	3	8	7	6	2	15
	Brain	2	85	85	5	82	88
	Kidney	5	25	18	14	15	48
	Liver	5	21	17	10	9	33

	Muscle	5	62	67	25	20	82
	Placenta	4	33	40	15	10	43
Se:Hg	Blubber	3	0.2	0.2	0.04	0.1	0.2
	Brain	2	1.4	1.4	1.0	0.7	2.1
	Kidney	5	1.4	1.6	0.4	0.9	1.8
	Liver	5	1.0	0.9	0.4	0.5	1.6
	Muscle	5	0.9	1.0	0.2	0.6	1.1
	Placenta	4	7.3	7.1	0.4	7.0	7.8

Table 9 Concentrations (ng/g ww.) of total mercury (THg), concentrations of total selenium (TSe), concentrations of methylmercury (MeHg), percentage of methylmercury out of total mercury (%MeHg), and selenium to mercury (Se:Hg) molar ratios from the brains of Saimaa ringed seals by age group.

Age class	Chemical	n	Mean	Median	St.dev	Min	Max
Pre-weaned pups	THg	4	140	111	87	72	266
	TSe	4	106	107	19	84	125
	MeHg	4	154	138	85	68	270
	%MeHg	4	114	100	32	95	161
	Se:Hg	4	2.5	2.3	1.4	0.9	4.4
Weaned pups	THg	3	433	271	345	199	830
	TSe	3	157	166	30	123	181
	MeHg	3	450	320	336	198	831
	%MeHg	3	106	100	11	99	118
	Se:Hg	3	1.3	1.6	0.7	0.5	1.7
Subadults	THg	3	530	462	158	417	710
	TSe	3	136	131	10	130	147
	MeHg	3	635	552	164	530	824

	%MeHg	3	121	119	6	116	127
	Se:Hg	3	0.7	0.8	0.2	0.5	0.8
Adults	THg	2	3710	3710	750	3180	4240
	TSe	2	1202	1202	605	774	1630
	MeHg	2	1279	1279	738	757	1800
	%MeHg	2	37	37	27	18	57
	Se:Hg	2	0.8	0.8	0.3	0.6	1.0

Table 10 Dry weight adjusted concentrations (ng/g) of total mercury (THg), concentrations of total selenium (TSe), concentrations of methylmercury (MeHg), percentage of methylmercury out of total mercury (%MeHg), and selenium to mercury (Se:Hg) molar ratios from the brains of Saimaa ringed seals by age group.

Age class	Chemical	n	Mean	Median	St.dev	Min	Max
Pre-weaned pups	THg	4	116	95	70	58	215
	TSe	4	88	89	18	67	107
	MeHg	4	127	117	68	55	218
	%MeHg	4	94	85	24	75	129
	Se:Hg	4	2.0	2.0	1.1	0.7	3.5
Weaned pups	THg	3	325	204	153	153	620
	TSe	3	118	124	21	94	136
	MeHg	3	338	241	249	152	621
	%MeHg	3	80	76	8	75	89
	Se:Hg	3	1.0	1.2	0.5	0.4	1.3
Subadults	THg	3	372	304	126	296	517
	TSe	3	95	95	1	94	95
	MeHg	3	447	386	134	353	600
	%MeHg	3	85	85	8	77	93

	Se:Hg	3	0.5	0.5	0.1	0.3	0.6
Adults	THg	2	2922	2922	593	2502	3341
	TSe	2	947	947	478	609	1284
	MeHg	2	1006	1006	580	597	1416
	%MeHg	2	29	29	22	14	45
	Se:Hg	2	0.6	0.6	0.2	0.5	0.8

63

References

Cáceres-Saez, Iris, Natalia A. Dellabianca, R. Natalie, P. Goodall, H. Luis Cappozzo, and Sergio Ribeiro Guevara. 2012. "Mercury and Selenium in Subantarctic Commerson's Dolphins (Cephalorhynchus c. commersonii)." *Biological Trace Element Research* 151 (2): 195-208.

Dehn, Larissa-A., Gay G. Sheffield, Erich H. Follmann, Lawrence K. Duffy, Dana L. Thomas, Gerald R. Bratton, Robert J. Taylor, and Todd M. O'Hara. 2005. "Trace Elements in Tissues of Phocid Seals Harvested in the Alaskan and Canadian Arctic: Influence of Age and Feeding Ecology." *Canadian Journal of Zoology* 83 (5): 726-746.

Dietz, R., F. Riget, and E. W. Born. 2000. "An Assessment of Selenium to Mercury in Greenland Marine Animals." (The Science of the Total Environment) 245: 15-24.

National Center for Biotechnology Information. 2021a. *PubChem Compound Summary for CID 23931, Mercury*. National Centre for Biotechnology Information. 4 15. Accessed 4 10, 2021. https://pubchem.ncbi.nlm.nih.gov/compound/Mercury.

National Center for Biotechnology Information. 2021b. *PubChem Compound Summary for CID 6326970, Selenium.* National Center for Biotechnology Information. 4 15. Accessed 4 15, 2021. https://pubchem.ncbi.nlm.nih.gov/compound/Selenium.
